# Whole-body vibration decreases delayed onset muscle soreness following eccentric exercise in elite hockey players: a randomised controlled trial

**DOI:** 10.1186/s13018-021-02760-4

**Published:** 2021-10-12

**Authors:** Harold Akehurst, John E. Grice, Manuela Angioi, Dylan Morrissey, Filippo Migliorini, Nicola Maffulli

**Affiliations:** 1 Department of Trauma and Orthopaedics, Great Western Hospital, Swindon, UK; 2 Centre for Sports and Exercise Medicine, William Harvey Research Institute, Queen Mary University of London, London, UK; 3 Department of Orthopaedic, Trauma, and Reconstructive Surgery, RWTH Aachen University Hospital, Pauwelsstraße 30, 52074 Aachen, Germany; 4 Department of Trauma and Orthopaedics, University of Salerno School of Medicine, Surgery and Dentistry, Salerno, Italy

**Keywords:** Delayed onset muscle soreness, DOMS, Whole-body vibration, WBV, Elite athletes, Eccentric exercise

## Abstract

**Background:**

Delayed onset muscle soreness (DOMS) is a common non-structural muscle injury which can disrupt training and impair performance in elite athletes. Vibration therapy reduces inflammation and improves neuromuscular efficiency, leading to reductions in pain and stiffness, and may be effective for the prevention or treatment of DOMS. However, the effect of whole-body vibration (WBV) used after sport in elite athletes has not been reported.

**Methods:**

A randomised, controlled trial was performed. Participants were elite (national or international level) hockey players and underwent an eccentric exercise protocol previously shown to produce clinical DOMS. After exercise, one group underwent static stretching with WBV therapy, and the other performed stretching only. Baseline and serial post-exercise pain scores and measurements of quadriceps tightness were obtained.

**Results:**

Eleven participants were recruited into each study arm. There were no significant differences in baseline group characteristics. Participants receiving WBV had significant reductions in both pain (*p* = 0.04) and quadriceps tightness (*p* = 0.02) compared with stretching only.

**Conclusions:**

Post-exercise WBV is effective in elite hockey players to reduce DOMS after eccentric exercise. Elite athletes in multi-sprint sports are at risk of DOMS during training and competition, and its reduction could contribute to reduced injury risk and improved performance. This treatment modality is favourable because it can be incorporated with minimal disruption into the recovery section of existing training regimes. These findings may also be extrapolated to other multi-sprint sports.

## Background

Delayed onset muscle soreness (DOMS) was described in 1900 by Hough, who reported soreness and loss of function developing around 12 h after exercise and lasting several days, which he attributed to muscle fibre damage and inflammation [1]. This description remains remarkably consistent with current understanding, which adds a number of theories for the specific mechanisms of DOMS, including lactic acid, muscle spasm, connective tissue damage, muscle damage, inflammation and enzyme efflux [2–5]. Non-structural injuries such as DOMS account for 70% of muscle injuries in soccer players, and more than 50% of days absent from sport activity and training [6, 7]. Significant DOMS persists for at least 72 h following professional soccer matches, resulting in increased training injury risk and impacting on performance [8]. DOMS is more severe in untrained individuals or following an unaccustomed activity in athletes and may result from excessive and prolonged eccentric muscle contractions [2, 9]. Its prevention and treatment therefore have significant implications both for performance at the elite level and for sustaining participation at the recreational level. A number of methods of prevention or treatment of DOMS have been investigated, including massage [10], cryotherapy [11], active recovery [12], homeopathy [13], acupuncture [14], TEN [15], ultrasound [16], non-steroidal anti-inflammatories [17], steroids [18], vitamin C and antioxidants [19], but consensus support for any single method remains to be established [2] (Table 1). Table 2 provides serial measurements of pain and tightness abrasion therapy which has been proposed in a number of applications in sport. The acute effects of vibration include reduced inflammation, reduced pain, increased flexibility, increased neuromuscular efficiency and increased strength [20–25]. Vibration therapies may be broadly categorised by whether they are applied locally or to the whole body (WBV), and whether they are used before, during or after exercise; specific characteristics including the frequency of vibration and the number and duration of applications vary widely. Vibration therapy has been studied extensively in the specific context of DOMS [26]. It has been proposed that vibration may optimise muscle performance by synchronising motor unit activity and increasing blood flow and that this may prevent mechanical sarcoma disruption and in turn reduce DOMS [26–28]. The overall picture is encouraging, with meta-analysis finding vibration effective for prevention or treatment of DOMS [29, 30]. Therapies studied include those provided before [31–36] and after [9, 20, 35, 37–49] exercise; in athletes [36, 49], untrained [9, 31–35, 37, 38, 40, 42, 45, 47, 48] and recreationally active [20, 39, 41, 43, 44, 46] participants; and using local vibration [20, 31, 32, 34, 35, 37, 38, 40, 45, 48, 49] and WBV [9, 33, 36, 39, 41–44, 46, 47]. However, the specific case of WBV used post-exercise in elite athletes has not previously been examined. This may represent a significant knowledge gap, as the development of DOMS does vary with level of training, and corresponding variation may be anticipated in therapeutic effects of WBV [50, 51]. Hockey is a multi-sprint sport with similar movements to soccer, yet additionally has multiple repetitions of eccentric exercises such as lunge tackling. Field hockey players are particularly at risk of DOMS, which can harm performance [52]. Elite hockey players competing in sequential games in a tournament are less able to sprint in the second and third games, with DOMS being a probable contributing factor [52].

**Table 1:** Baseline group characteristics

Endpoints	WBV	Stretch only
*Sex*		
Male	7 (64%)	7 (64%)
Female	4 (36%)	4 (36%)
*Age (years)*	26	27
*Competition level*		
International	6 (55%)	5 (45%)
National	5 (45%)	6 (55%)
*Usual training (hours/week)*		
2–5	0 (0%)	1 (9%)
5–10	9 (82%)	9 (82%)
> 10	2 (18%)	1 (9%)
*Body mass index (kg/m*^*2*^*)*		
20–25	11 (100%)	10 (91%)
> 25	0	1 (9%)

**Table 2:** Serial measurements of pain and tightness

Endpoints	Baseline	Follow-up					*P* value^†^
		Day 0	Day 1	Day 2	Day 3	Day 7	
*Pain (VAS)*							
Stretch only	0 ± 0	27.2 ± 20.0	29.8 0 ± 18.2	25.3 ± 23.2	17.5 ± 23.2	1.3 ± 4.2	0.04
WBV	0 ± 0	32.1 ± 21.1	27.6 ± 19.1	11.2 ± 11.6	2.6 ± 5.1	0 ± 0	
*Tightness (cm)*							
Stretch only	12.5 ± 4.2	13.6 ± 4.0	15.0 ± 4.0	15.3 ± 4.9	15.0 ± 4.8	12.9 ± 4.3	0.02
WBV	13.8 ± 3.5	14.1 ± 4.3	14.7 ± 3.7	14.5 ± 3.4	13.6 ± 2.9	12.4 ± 3.3	

^†^For difference in area under the curve

The primary aim of this study was to assess whether WBV-administered post-eccentric exercise is more effective to reduce DOMS in elite hockey players than static stretching. The objectives were to produce clinical DOMS using a validated exercise protocol, to obtain serial pain and functional measurements, and to compare these between treatment groups.

## Materials and methods

### Ethics

Approval was obtained from the Queen Mary Research Ethics Committee (QMREC2012/50). Participants were provided written and verbal information about participation. All subjects gave informed consent to participate.

### Population, randomisation and sampling

Elite (National league or International) field hockey players above the age of 18 were invited to volunteer for the study. Both male and female hockey players were invited. Exclusion criteria were pre-existing injury, systemic disease, muscle disease or previous surgery to the lower limb and non-provision of consent.

Using a closed envelope method, participants were randomly allocated to the intervention or control group. Randomisation, enrolment and assignment were performed by one of the investigators (JG). All players were prescribed a standard eccentric exercise protocol [9] and underwent the same testing procedure at 24, 48, 72 h and at 1 week. Only the intervention group underwent the WBV protocol.

Other active recovery work was prevented alongside massage and cryotherapy. Additionally, the use of compression clothing or anti-inflammatories was forbidden in the post-exercise period to avoid bias. Consequently, the hockey off-season period was identified as an appropriate period for testing. Data were collected at Nuffield Health Gym, Surbiton, UK, and Fareham Leisure Centre, Fareham, UK.

#### Sample size

Sample size was calculated based on a primary outcome of difference between areas under the curve (AUC) of serial pain visual analogue score (VAS) from day 0 to day 7 post-exercise, anticipating a difference of 5 points per day corresponding with a difference in AUC of 40 point-days. Based on a standard deviation for the AUC (derived from the reported standard deviations in pain VAS following a comparable exercise regime) [9], two equal groups of *n* = 8 were calculated to provide 90% power at the 0.05 level of significance.

### Exercise and recovery protocols

The exercise commenced with a warmup of 5 min on a seated bicycle (Cybex, Minnesota, USA) at 70 revolutions per minute with zero resistance. Following the warm-up, quadriceps tightness was assessed by measuring the distance between heel and buttocks (cm) during a maximal stretch of the muscle group without using the hand to aid the stretch. All participants were asked their pre-exercise VAS perceived pain in the quadriceps area. The 1 repetition maximum (1 RM) was ascertained to 5 kg accuracy by attempting 1 repetition of a knee extension using a seated knee extension machine fixed at 40 kg (Cybex, Minnesota, USA) and, if successful, attempt 1 repetition at increments of 5 kg until the exercise was not possible. The value below this failure level was taken as the 1 RM. Ideally, the 1 RM would be found at a preceding date. However, because of time, travel and cost constraints, this was not always achievable.

The eccentric exercise protocol was based on that of Rhea et al., which produced clinical DOMS with peak pain VAS in a static stretch control group of 70/100 at 48 h post-exercise [9]. Eccentric exercises were performed on a seated knee extension machine consisting of negative repetition knee extensions, 4 × 10 repetitions at 60% 1RM unless prevented by fatigue. The exercise was standardised by timing the eccentric phase of the exercise to be completed in 6 s, and the concentric phase performed as quickly as possible. On the final set, the participant was encouraged to continue the exercises to exhaustion.

### WBV protocol group

**Fig. 1:**
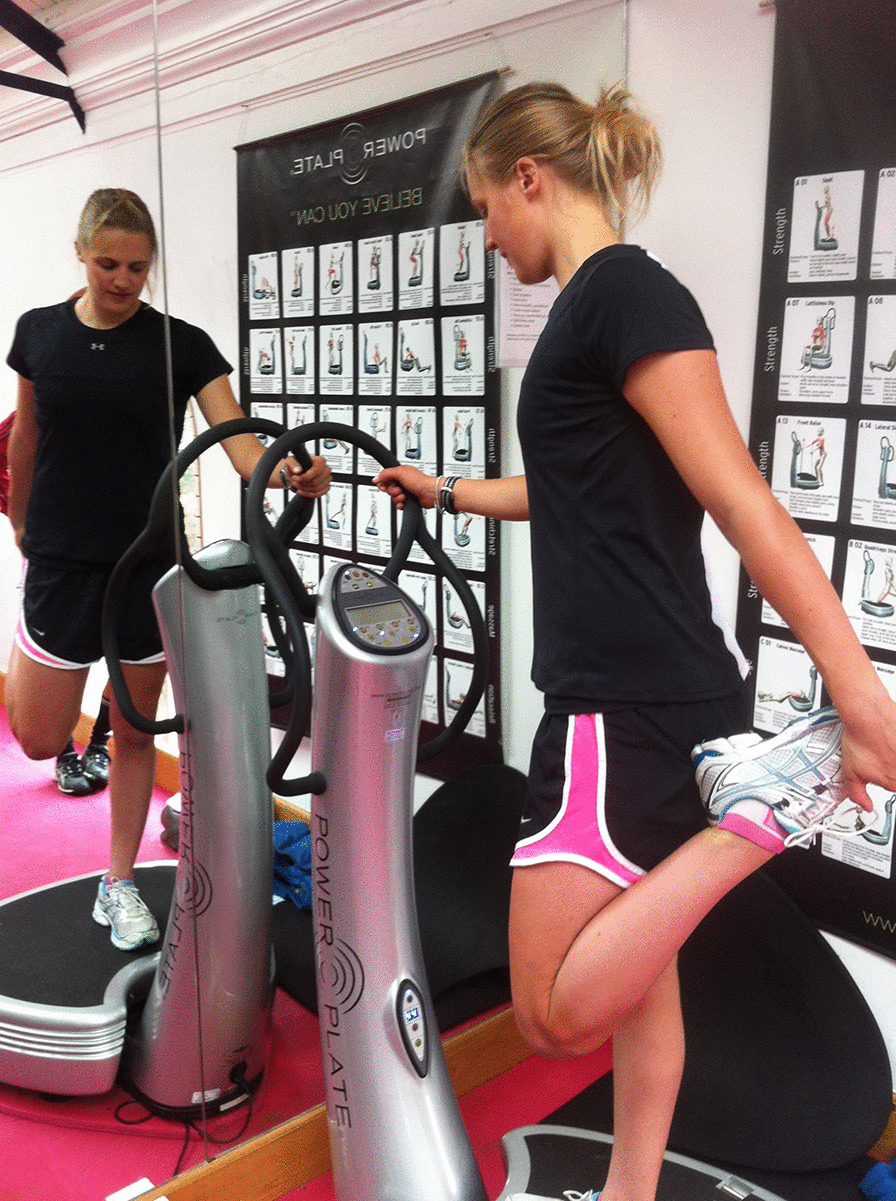
Quadriceps stretch during WBV therapy

Low-frequency vibrations (30 Hz) at amplitude of 4 mm were used. While WBV has been studied at frequencies from 12 to 50 Hz and amplitudes from 1 to 5 mm, these parameters were chosen in order to facilitate comparison with the largest number of other studies, which employ the 30–35 Hz range [9, 30, 47]. Although the acute effects of different frequencies of WBV (20 and 40 Hz) on muscle performance and flexibility have been directly compared, their impact on DOMS was not reported [24]. Each participant completed three sets of 2 min each, with 2 min rest between sets. This protocol (including rests between sets) was selected to reduce the level of fatigue from vibrations [24]. The first minute of each set was spent with 30 s stretching the right quadriceps (Fig. 1).

**Fig. 2:**
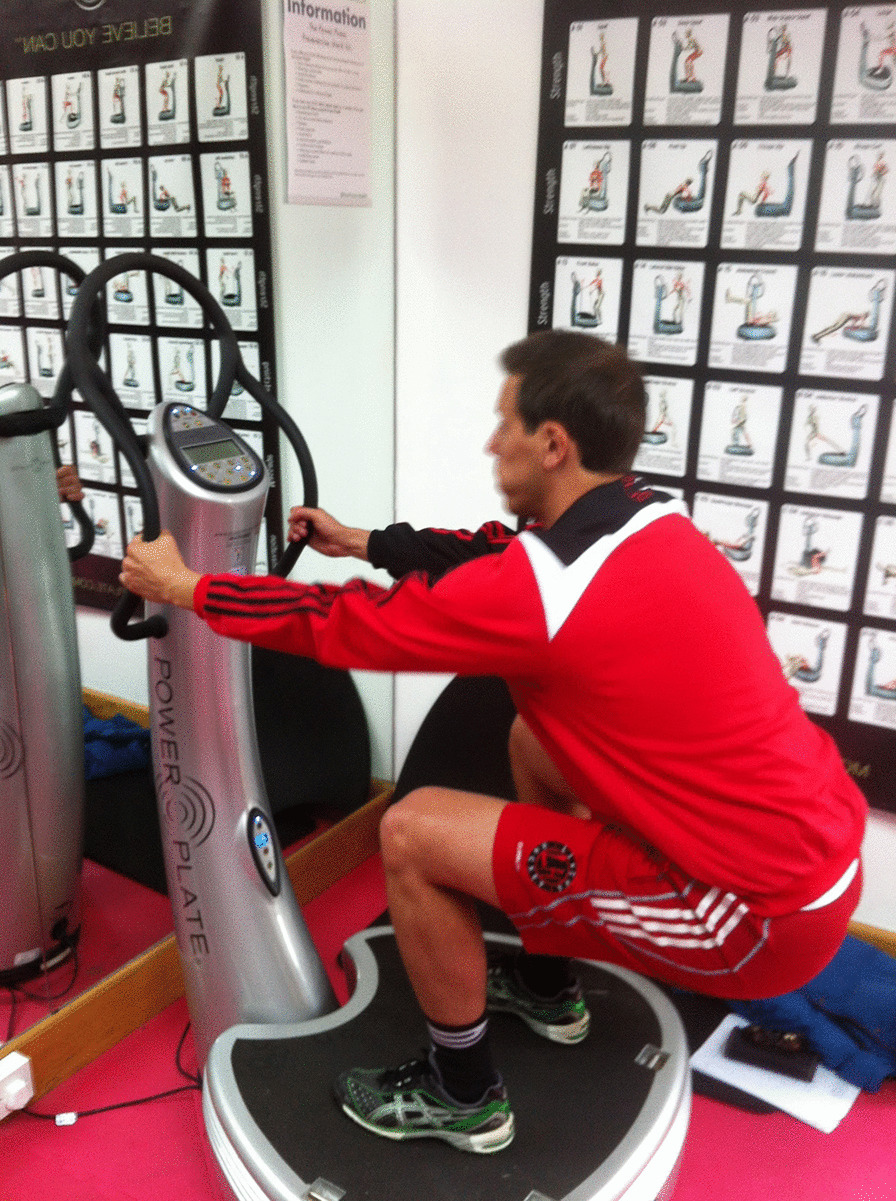
Squat position during WBV therapy

The second 30 s was spent stretching the left quadriceps. The second minute was spent in a squatting position with 90° knee flexion with the knees over the feet and the back held in a neutral position (Fig. 2). The protocol used a Pro5 Power Plate® (Power Plate International Ltd., London, UK).

### Stretching (control) group

The control group performed the stretches alongside the WBV group simultaneously, but in the absence of WBV. A quadriceps stretch was produced by the ipsilateral hand holding the foot and producing maximal knee flexion while extending the hip (Fig. 1).

### Visual analogue scale and quadriceps tightness

The participants documented their pain/soreness on a VAS ranging from 0 to 100 with 100 being maximal pain imaginable and 0 being no pain [53]. In the prone position, quadriceps tightness was measured as described above. Measurements were performed by a single investigator (JG). Anthropometry is usually very reliable, and independent reliability measurements were therefore not undertaken [54, 55].

### Statistical analysis

Data were stored in a spreadsheet and analysed in R version 3.4.3 (R Core Team, Vienna, Austria). Descriptive analysis was used to compare group characteristics. The area under the curve for serial pain VAS was calculated, and distributions were assessed with histograms and box plots to confirm Normal distribution. Group means were compared using the two-sample Welch t test. Serial quadriceps length measurements were expressed as proportions of the pre-exercise measurement and log-transformed before calculation of the area under the curve (to re-scale around 0). The distributions of areas under the curve were skewed, and therefore group medians were compared using the Wilcoxon rank-sum test.

## Results

**Fig. 3:**
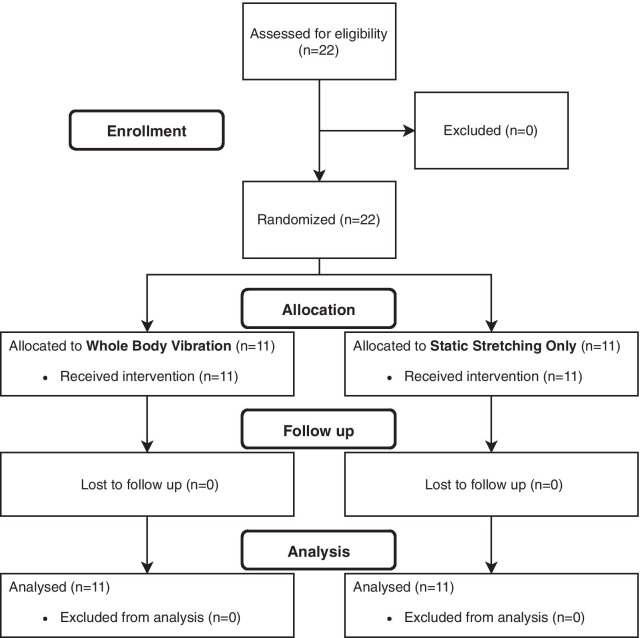
CONSORT diagram

A total of 22 participants (14 males and 8 females) were recruited and allocated to either the WBV or stretch-only control group between June and July 2012. Eleven participants were randomised to each group. The two groups demonstrated similar characteristics at baseline (Fig. 3).

### Participants

There were no statistically significant differences in age, playing ability, sex and training intensity between the groups.

**Fig. 4:**
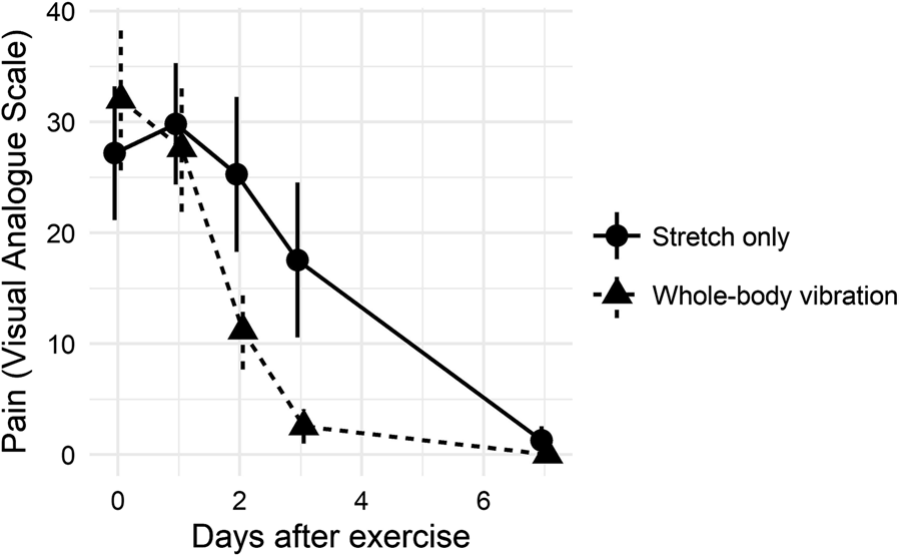
Post-exercise pain visual analogue score (VAS)

Serial pain VAS for stretch-only and WBV groups was compared (Table 2; Fig. 4). Mean areas under the curve were 115 (95% confidence interval 68–162) and 61 (95% CI 37–85) units for stretch-only and WBV groups, respectively (*p* = 0.04).

**Fig. 5:**
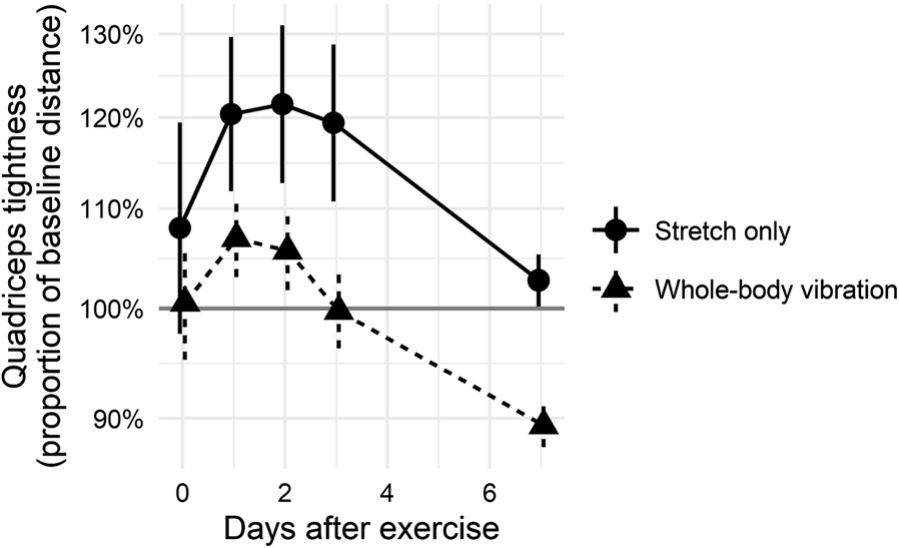
Post-exercise quadriceps tightness expressed as proportion of baseline distance between heel and buttock on maximal voluntary knee flexion

Serial quadriceps tightness measurements were also compared (Table 2; Fig. 5). Mean areas under the (log-transformed) curve were 0.92 (95% CI 0.05–1.78) and − 0.11 (95% CI − 0.39 to 0.18) units for stretch-only and WBV groups, respectively (*p* = 0.02).

All participants returned to normal training within 2 weeks.

## Discussion

We performed a randomised controlled trial of post-exercise WBV for the reduction in DOMS in elite hockey players. Both pain and muscle tightness were significantly improved after WBV in comparison with stretching alone. The WBV therapy regime did not require additional time spent on recovery above static stretching and therefore may be integrated into existing training programmes with minimal disruption.

Results from previous studies of post-exercise WBV for prevention of DOMS have recruited either recreational or non-athletes. Results have been equivocal with no significant improvement in pain reported by five randomised trials [41–44, 46]. However, improvements in pain were found by two other trials and other literature studies [9, 39, 47]. It is established that physiological responses to exercise including the development of DOMS are altered by training, and the novel use of elite athletes in the present study may partly explain our positive findings [50, 51]. Wide variation in treatment protocols (including timing and number of sessions, vibration frequency, etc.), control group selection, exercise protocols used to induce DOMS, and outcome measures used also underlie variation in results. For research purposes, consensus around measures of DOMS and gold standard control groups is highly desirable.

Other post-exercise studies of vibration therapy have employed local vibration rather than WBV. Results from these studies have been generally favourable [20, 37, 38, 40, 48, 49]. Comparisons of local vibration with WBV have found local vibration superior for lower limb flexibility, but no difference in jump performance [56, 57]. The effects of the two modalities on DOMS have not been directly compared and should be the subject of future studies.

This trial investigated post-exercise vibration therapy. Vibration therapy has been most often studied as a recovery modality, but favourable results have been reported by multiple studies of its pre-exercise use [32, 33, 36]. In a trial which compared the use of local vibration before or after exercise with a third control group, pre-exercise therapy produced greater reductions in pain and laboratory measures of muscle damage than post-exercise therapy [35]. Further investigation is required directly comparing pre- to post-exercise vibration therapy.

The influence on results of confounding factors is minimised by the use of randomised controlled trial methodology. In addition, the selection of stretching alone for the control group provides ‘real-world’ relevance, because stretching remains the most common recovery modality used by both recreational and elite athletes and is widely practised to reduce muscle pain [58]. These data are a relatively novel contribution to knowledge of the effects of post-exercise WBV therapy for prevention of DOMS in elite athletes. Blinding was impractical due to the nature of the intervention, and subjective VAS results may therefore be influenced by the placebo effect. Lack of blinding could also have influenced performance in quadriceps length testing, as well as introducing assessment bias in assessors. This could be minimised in future studies comparing related vibration therapy protocols, where blinding is more feasible. Reliability analysis of quadriceps length measurement was not undertaken, and measurement error is therefore also possible. However, the reliability of anthropometry is generally high [54, 55]. The training programme used has previously been shown to induce clinical DOMS, and therefore all participants completing the training programme were included in analysis without further confirmation or quantification of DOMS [9]. However, there is evidence that the same training program can result in variable stimuli to muscles in different individuals, and it is possible that the training programme did not produce ‘true’ DOMS in all participants [59]. Pain and stiffness are measures of function but are not direct evidence of the pathology of DOMS. Bias resulting from this effect is minimised by randomisation. Additionally, the delayed peak mean pain and loss of flexibility observed do suggest the presence of true DOMS. Future studies with adequate sample size may include subgroup analyses of participants with confirmed DOMS (based, for example, on laboratory testing or threshold clinical measurements). Sample size was relatively small although comparable with previous studies and necessarily limited by the availability of elite hockey players to participate [9, 20, 33, 38]. While the study was adequately powered, a greater sample size would be desirable for accurate estimation of effect sizes. It was not possible to enforce complete rest during the 7-day post-exercise observation period, and some participants may have performed limited active recovery through manual or physical occupations. Bias resulting from this is minimised by randomisation, but may have influenced results. As some variable physical activity is inevitable in ‘real’ settings, future studies should record such activity and consider incorporating it into the recovery protocol. The physiological differences between male and female athletes may make comparison between the two sexes inaccurate. However, most studies have demonstrated a similar level of muscle damage following eccentric exercise in both sexes [60]. Females can be more prone to muscle damage than males post-exercise [60, 61], but may recover quickly with an attenuated inflammatory response [60]. Due to time, travel and cost constraints, some participants were not able to attend an additional initial appointment to measure the 1RM, and this was done before beginning the eccentric exercise protocol. While this additional exertion may have had an impact on performance of the exercise protocol, its impact on the development of clinical DOMS or on treatment effects is not likely to be substantial.

## Conclusions

WBV can be used in elite athletes to reduce the signs and symptoms of DOMS post-eccentric exercise. This finding could be extrapolated to other multi-sprint sports. Future studies could be performed with larger numbers in a randomised controlled trial with better controls to compare the effect on athletes from various sporting modalities, with the addition of muscle biopsy to further assess histological evidence of DOMS.

## Data Availability

Neither ethical approval nor informed consent was sought for public deposit of data, which are therefore not made publicly available.

## Abbreviations

DOMS: Delayed onset muscle soreness
WBV: Whole-body vibration
AUC: Areas under the curve
VAS: Visual analogue score
RM: Repetition maximum
